# Spontaneous pregnancy after tracking ovulation during menstruation: A case report of a woman with premature ovarian insufficiency and repeated failure of *in vitro* fertilization

**DOI:** 10.3389/fmed.2022.994674

**Published:** 2022-12-08

**Authors:** Ye He, Wanlu Wang, Chunmei Wu, Wenwen Li, Hongjiang Liu, Zhaolian Wei

**Affiliations:** ^1^Department of Obstetrics and Gynecology, The First Affiliated Hospital of Anhui Medical University, Hefei, China; ^2^NHC Key Laboratory of Study on Abnormal Gametes and Reproductive Tract (Anhui Medical University), Hefei, China; ^3^Key Laboratory of Population Health Across Life Cycle (Anhui Medical University), Ministry of Education of the People's Republic of China, Hefei, China; ^4^Department of Obstetrics and Gynecology, The People's Hospital of Huaibei City, Huaibei, China

**Keywords:** premature ovarian insufficiency, repeated failure of *in-vitro* fertilization, spontaneous pregnancy, ovulation during menstruation, live birth

## Abstract

The diagnosis of premature ovarian insufficiency (POI) is devastating in women of reproductive age because of the small chance of spontaneous pregnancy. Here, we report a very rare case with POI and repeated failure of *in vitro* fertilization (IVF) where the final result was natural fertilization following guidance to have sexual intercourse during menstruation as ovulation was monitored. Estradiol valerate was used to increase the thickness of the endometrium and stop the menstrual bleeding. There was a serum level of 208.44 IU/L of human chorionic gonadotropin (HCG) 14 days after the ovulation. Later, a series of transvaginal ultrasounds also indicated a normal-appearing intra-uterine pregnancy. A healthy baby girl was delivered at term by means of cesarean section. Our report suggested that although the chance of spontaneous pregnancy is relatively low in patients with POI with repeated IVF failures, as long as ovulation does occur, even if it happens during menstruation, natural pregnancy is still worth trying with a series of proper and personalized treatments.

## Introduction

Premature ovarian insufficiency (POI) is a clinical syndrome defined as the loss of normal ovarian activity before the age of 40 (1). Approximately 1% of women under the age of 40 and 0.1% of women under the age of 30 develop POI (1). It is characterized by menstrual disturbances with raised gonadotrophins and low estradiol. The most frequent pattern of menstrual irregularity for POI is the gradual onset of menstrual cessation with either shortened, prolonged, or variable cycle length (2).

POI imposes a great challenge on women's fertility, given the cessation of ovarian function and the declining oocyte quality. Women with POI should be informed that there is only about a 5% chance of getting pregnant spontaneously (3) with no interventions that can reliably increase ovarian activity and natural conception rates (4). *In vitro* fertilization (IVF) with autologous oocytes has been recommended as a treatment for women with POI when the residual ovarian reserve is sufficient for ovarian stimulation (5). However, multiple consecutive IVF cycles to achieve conception is usually physically and mentally exhausting. Egg donation by IVF is often the final solution, but many women cannot accept it and would need a long time for psychological construction (1, 6). Furthermore, oocyte donation is not permitted in many countries because of ethical problems. A 2019 systematic review including 15 studies summarized the pregnancy following the diagnosis of POI (4). It emphasized an important point that even though pregnancies without donor egg treatment are unusual, however, they do occur, and clinicians should not be too hasty to label young women with POI as beyond hope (4). Therefore, natural pregnancy is still worth trying for patients with POI and the induction of ovulation and seizing the timing of ovulation are critical.

Here, we report a rare case of POI with repeated failed IVF but got naturally conceived with the guidance of maintaining sexual life during menstruation because of the monitored ovulation.

## Case description

A 32-year-old woman with a POI diagnosis presented to our fertility clinic on August 2021 because of the desire to get pregnant but with repeated failures of IVF. The patient denied a family history of POI, infertility, autoimmune disease, or a history of chemotherapy or radiation therapy. The patient had menarche at the age of 13, with regular menstruation until the age of 30. The couple's chromosomes were both normal.

The patient had one natural pregnancy in 2018 at the age of 28 before the POI occurred; however, the embryo stopped developing in week 8 because of chromosome 16 trisomy. At that time, her menstrual cycle was regular (cycle length of about 28–30 days). After this miscarriage, the patient received 1-year treatment of traditional Chinese medicine to activate blood, remove stasis, and promote circulation (which does not affect ovarian function) but did not experience a successful pregnancy.

On May 2020, at the age of 30, the patient's menstruation started getting irregular without expectation. Her menstrual cycle length was shortened to 20–22 days, and her menstrual volume was reduced significantly. The blood testing showed that serum follicle-stimulating hormone (FSH) was 40.00 IU/L, estrogen was 114.50 pmol/L, progesterone was 1.31 ng/ml, and anti-Mullerian hormone (AMH) was 0.18 ng/ml. The transvaginal ultrasound (TVS) indicated that the bilateral ovarian volume was significantly smaller than normal ovaries with few antral follicles. The diagnosis of POI was made. The couple then went to a fertility clinic in Shanghai (China) seeking infertility treatment by IVF. A total of five times ovulation induction with four IVF were conducted from 2020 to 2021 with either micro-stimulation or natural cycle protocol. For each IVF cycle, the patient got one or two oocytes. Finally, five oocytes and two embryos were received. However, the two transplantations failed.

On August 2021, the patient visited our fertility center for consultation. We asked the patient to have estradiol valerate and progesterone treatment (hormone replacement therapy) for 2 months. If menstruation happens during the treatment, she should revisit us to check the ovarian reserve.

On 8 September 2021, the patient revisited our center on the 3rd day of menstruation. The TVS indicated a 20^*^20-mm cyst on the left ovary, and the thickness of the endometrium was 5.7 mm (Supplementary Table 1 and Supplementary Figure 1). The hormone test showed that the serum estrogen was 1,316 pmol/L and the FSH was 5.23 IU/L. All these suggested that this cyst should be a matured follicle. Therefore, we strongly suggested to the patient that they should seize this chance to get pregnant naturally. A guided sexual intercourse was scheduled on 9 September 2021 (day 4 of the menstruation) and 11 September 2021 (day 6 of the menstruation). The oral estradiol valerate (2 mg twice daily; Progynova, Baye) was given to help increase the thickness of the endometrium. On day 7 of the menstruation, the re-checked TVS indicated that the cyst disappeared and the thickness of the endometrium was 7.4 mm (Supplementary Table 1 and Supplementary Figure 2). The ovulation was inferred to occur between day 4 and day 6 of the menstruation. The estradiol valerate was continuously used (2 mg twice daily; Progynova, Baye). Luteal phase support was provided with the dydrogesterone tablets (10 mg twice daily; Duphaston, Abbott) for 14 days to prevent luteal phase defect.

Two weeks later, the blood testing indicated an increased level of human chorionic gonadotropin (HCG) (208.44IU/L) and progesterone (111.53IU/L), and a clinical pregnancy was diagnosed. Because of the slight bleeding, HCG (2,000 iu, every other day, 30 days) was applied, and dydrogesterone (10 mg twice daily, 60 days) and estradiol valerate (2 mg twice daily, 15 days) were continued.

**Figure 1:**
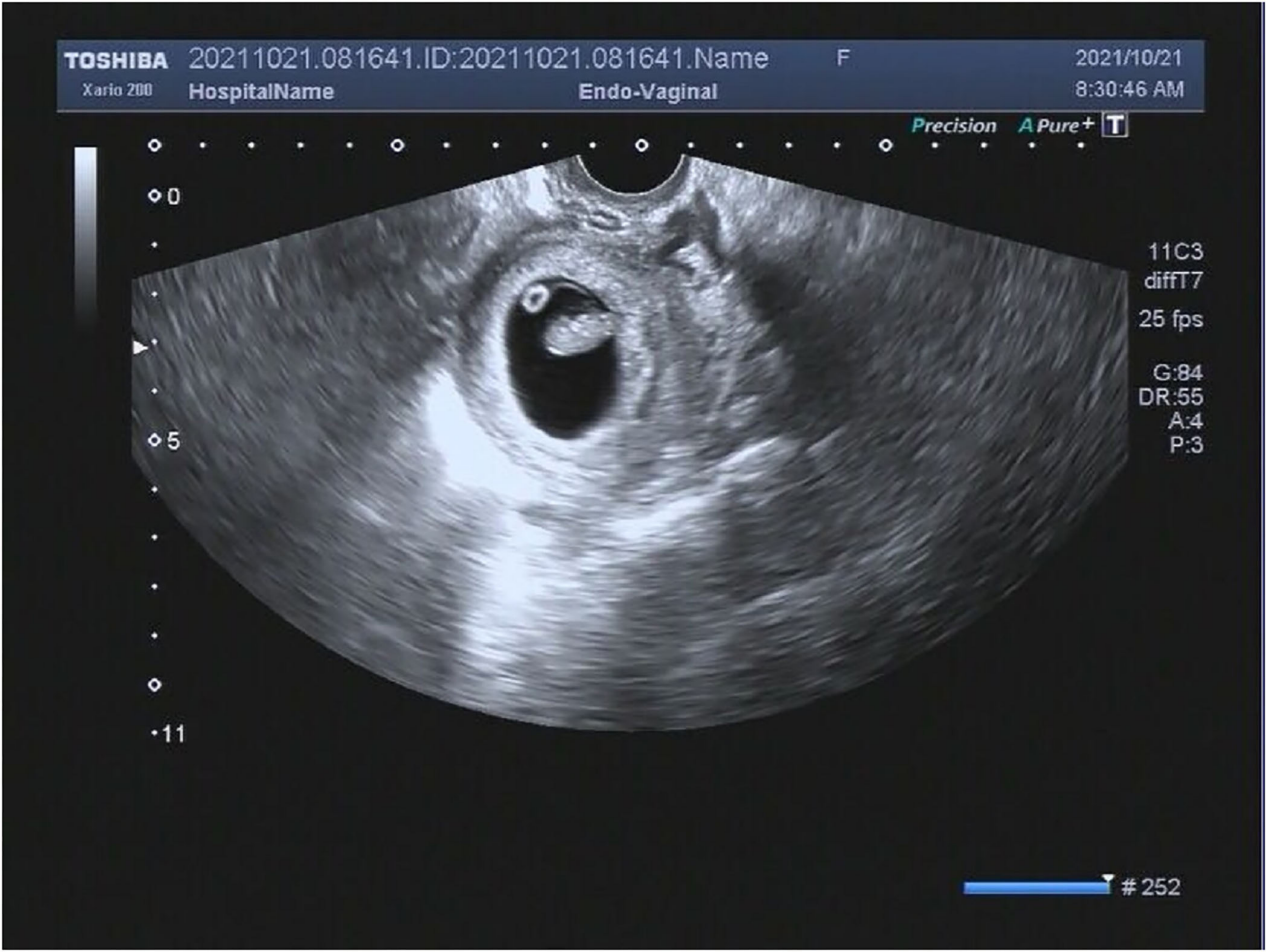
Transvaginal sonography of 8 weeks of gestation of pregnancy.

One month later, on 21 October 2021, TVS confirmed the intra-uterine pregnancy with a gestational sac of 36^*^26 mm, a yolk sac of 3 mm, and a crown-hip length of 15.7 mm with a visible heartbeat (Figure 1).

On 21 May 2022, a healthy baby girl weighing 3,345 grams was born by the cesarean section at 39 weeks and 1 day of the pregnancy, and the APGAR score for the baby was 10.

Written informed consent for the publication of this case report was obtained. The non-identifiable ultrasound image is included in the case report; however, approval for publication was still obtained.

## Discussion

The diagnosis of POI can be devastating for many women because of the difficulty in getting pregnant, even with assisted reproductive technology. The current case describes a 32-year-old woman with POI and repeated failure of IVF; she finally spontaneously conceived by following guidance to have sexual intercourse during menstruation because of the ovulation that occurred at this specific time point with the treatment of estradiol valerate.

Ovulation normally happens approximately on day 14 of menstruation; however, it is often ceased and/or particularly irregular in patients with POI. Spontaneous pregnancies are therefore extremely rare for patients with POI. Different from menopause, ovarian failure is not permanent, and ovarian activity may still occur in women with POI, especially in the early stage of the condition (7, 8), which still gives the possibility for spontaneous conception. In the current case, ovulation happened during days 4–6 of the menstruation may be because of the high FSH level and, therefore, the shortened menstrual cycle length for the patient.

To our knowledge, this is the first to report the case of a patient with POI who got conceived naturally by monitoring ovulation during menstruation and treating with estradiol valerate. Several previous studies have reported spontaneous pregnancy cases in patients with POI (9–11). In 2018, Calik-Ksepka et al. reported the case of a 27-year-old woman with POI treated with hormone replacement therapy, who, 6 months after diagnosis, conceived spontaneously (9). Later in 2020, Gu and Xu described a successful spontaneous pregnancy in a woman with POI and 10 years of amenorrhea (10). In the same year, Ayers CD and Carlson KS reported that a woman with POI conceived spontaneously during breastfeeding after the first pregnancy with donor eggs through IVF (11). However, all these cases were patients with amenorrhea for a long time who conceived spontaneously and unexpectedly after diagnosis without monitoring ovulation. Some patients with POI, especially those in the early stage, would still present with regular or irregular menstrual cycles (12). For those patients, seizing the timing of ovulation is very important for pregnancy naturally. In the current case, a matured follicle was tracked on day 3 of the menstrual cycle, and we proposed the patient have sexual intercourse during this time window. A series of treatment were provided: Estradiol valerate was used to increase the thickness of the endometrium and hinder the bleeding shortly. After the ovulation confirmed, progesterone and HCG was also administrated to support the luteal function in the first trimester and increase the stability of embryo development. The limitation of the treatment was that the pregnancy rate was not guaranteed because POI *per se* was associated with a decreased oocyte quality and low spontaneous pregnancy chance.

Although this is a rare case, the tailored strategy and the whole treatment progress for the current case to get pregnant naturally are of clinical significance. For patients with POI with repeated IVF failures, though the chance of getting spontaneous pregnancy is relatively low, as long as ovulation does occur and even if it is during menstruation, natural pregnancy is still worth trying with a series of proper and personalized treatments.

## Data availability statement

The raw data supporting the conclusions of this article will be made available by the authors, without undue reservation.

## Ethics statement

Written informed consent was obtained from the individual(s) for the publication of any potentially identifiable images or data included in this article.

## Author contributions

YH and WW drafted the manuscript. CW provided interpretive advice to the manuscript. WL and HL helped tease the history of the case report. ZW was involved in the acquisition of the case in the clinic and helped draft the manuscript. All authors have reviewed and approved the final manuscript version.

## Supplementary material

Supplementary Table 1 The sheet describes the whole process of monitoring ovulation under transvaginal sonography (English version).

Supplementary Figure 1 The original record of monitoring a dominant follicle under transvaginal ultrasound on the day 3 of the menstruation.

Supplementary Figure 2 The original record indicated the follicle ovulated on day 7 of the menstruation.

## References

[1] Webber L Davies M Anderson R Bartlett J Braat D Cartwright B. ESHRE guideline: management of women with premature ovarian insufficiency. Hum Reprod. (2016) 31:926–37. 10.1093/humrep/dew027 27008889

[2] Ivovic M Marina L Tancic-Gajic M Arizanovic Z Stankovic M Cirkovic A. Menstrual cycle characteristics in women with premature ovarian insufficiency. Endocr Abstr. (2019) 63:325. 10.1530/endoabs.63.P325

[3] Nelson LM. Clinical practice. Primary ovarian insufficiency. N Engl J Med. (2009) 360:606–14. 10.1056/NEJMcp0808697 19196677 PMC2762081

[4] Fraison E Crawford G Casper G Harris V Ledger W. Pregnancy following diagnosis of premature ovarian insufficiency: a systematic review. Reprod Biomed Online. (2019) 39:467–76. 10.1016/j.rbmo.2019.04.019 31279714

[5] Chae-Kim JJ Gavrilova-Jordan L. Premature ovarian insufficiency: procreative management and preventive strategies. Biomedicines. (2018) 7:2. 10.3390/biomedicines7010002 30597834 PMC6466184

[6] Bagheri M Jafarabadi M Vasegh Rahimparvar SF Nourbala AA Behboodi Moghadam Z. Concerns of infertile women candidates for egg donation: a qualitative study. J Family Reprod Health. (2020) 14:21–31. 10.18502/jfrh.v14i1.3784 32863835 PMC7428411

[7] Chon SJ Umair Z Yoon MS. Premature ovarian insufficiency: past, present, and future. Front Cell Dev Biol. (2021) 9:672890. 10.3389/fcell.2021.672890 34041247 PMC8141617

[8] Cox L Liu JH. Primary ovarian insufficiency: an update. Int J Womens Health. (2014) 6:235–43. 10.2147/IJWH.S37636 24591848 PMC3934663

[9] Calik-Ksepka A Grymowicz M Bronkiewicz W Urban A Mierzejewski K Rudnicka E. Spontaneous pregnancy in a patient with premature ovarian insufficiency - case report. Prz Menopauzalny. (2018) 17:139–40. 10.5114/pm.2018.78560 30357029 PMC6196780

[10] Gu Y Xu Y. Successful spontaneous pregnancy and live birth in a woman with premature ovarian insufficiency and 10 years of amenorrhea: a case report. Front Med. (2020) 7:18. 10.3389/fmed.2020.00018 32118005 PMC7018703

[11] Ayers CD Carlson KS. Spontaneous pregnancy in the setting of primary ovarian insufficiency and breastfeeding: does immunosuppression play a role? Am J Case Rep. (2020) 21:e926980. 10.12659/AJCR.926980 33127872 PMC7643410

[12] Piedade KC Spencer H Persani L Nelson LM. Optimizing fertility in primary ovarian insufficiency: case report and literature review. Front Genet. (2021) 12:676262. 10.3389/fgene.2021.676262 34249096 PMC8261244

