# Supplementary material for: Spontaneous pregnancy after tracking ovulation during menstruation: A case report of a woman with premature ovarian insufficiency and repeated failure of in vitro fertilization
Source: Front Med (Lausanne). 2022 Dec 8;9:994674. doi: 10.3389/fmed.2022.994674 (PMC9772434; doi:10.3389/fmed.2022.994674)
Supplement: Supplementary Table 1 — The sheet describes the whole process of monitoring ovulation under transvaginal sonography (English version). [file Table_1.DOCX]

| Date/The Day of the Menstruation | 2022.09.08/D3 of the Menstruation | 2022.09.12/D7 of the Menstruation |  |  |
| --- | --- | --- | --- | --- |
| Right Ovary(mm) | 21*17 | 20*16 |  |  |
| Follicle (mm) |  |  |  |  |
| Left Ovary(mm) | 33*29 | 35*30 |  |  |
| Follicle (mm) | A 20*20mm cyst is seen in the left ovary | The follicle has been ovulated |  |  |
| Uterus (mm) | Retroverted and the thickness is 34mm | Retroverted and the thickness is 34mm |  |  |
| Endometrium (mm) | 5.7 | 7.4 |  |  |
| Type of the Endometrium | Menstruation | B |  |  |
| CC |  |  |  |  |
| LE |  |  |  |  |
| HMG |  |  |  |  |
| HCG |  |  |  |  |
| Estradiol Valerate | 2 mg twice daily; Progynova, Baye | 2 mg twice daily; Progynova, Baye |  |  |
| Dydrogesterone |  | 10 mg twice daily; Duphaston, Abbott |  |  |
| Other Medicine |  |  |  |  |
| Urine LH |  |  |  |  |
| Other Description |  |  |  |  |
| The Clinical Diagnosis and Suggestions | 1. Have sexual life on 9th Sep and 11th Sep; 2. Check the ovulation on 12th Sep or 13th Sep | Test urine HCG 14 days later |  |  |

**Supplementary Table 1 The sheet describes the whole process of monitoring ovulation under transvaginal sonography (English version).**

The First Affiliated Hospital of Anhui Medical University

Reproductive Medicine Center for Monitoring Ovulation

Name: xxx Sex: Female Age:32 years

In the Day of the Menstruation Clinical Diagnosis: POI

Abbreviations: CC: clomiphene citrate; HMG: human menopausal gonadotropin; HCG: human chorionic gonadotropin; LH: Luteinizing Hormone.
