# Supplementary figures and images for: Spontaneous pregnancy after tracking ovulation during menstruation: A case report of a woman with premature ovarian insufficiency and repeated failure of in vitro fertilization
Source: Front Med (Lausanne). 2022 Dec 8;9:994674. doi: 10.3389/fmed.2022.994674 (PMC9772434; doi:10.3389/fmed.2022.994674)

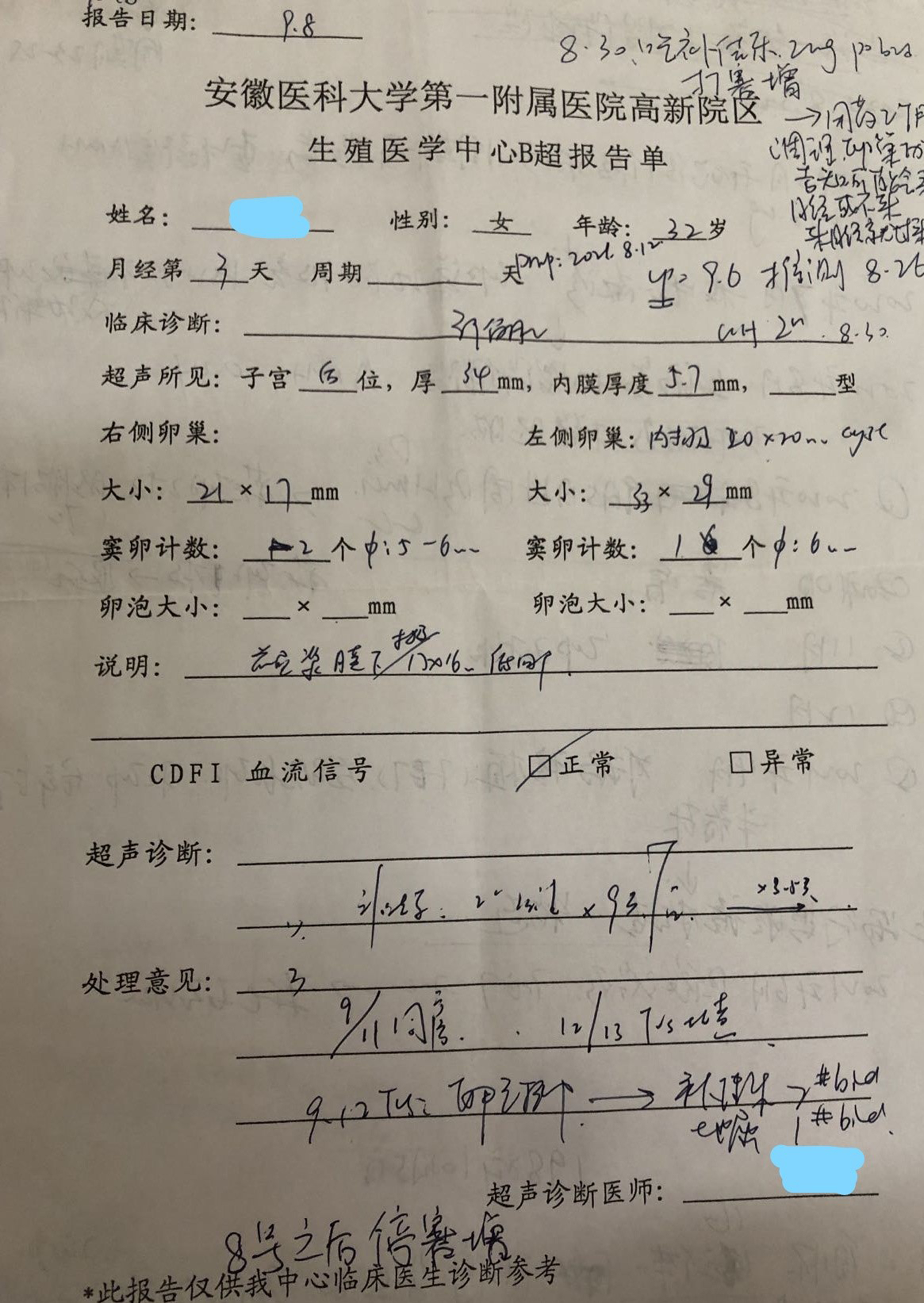

Supplement: Supplementary Figure 1 — The original record of monitoring a dominant follicle under transvaginal ultrasound on the day 3 of the menstruation. [file Image_1.JPEG]

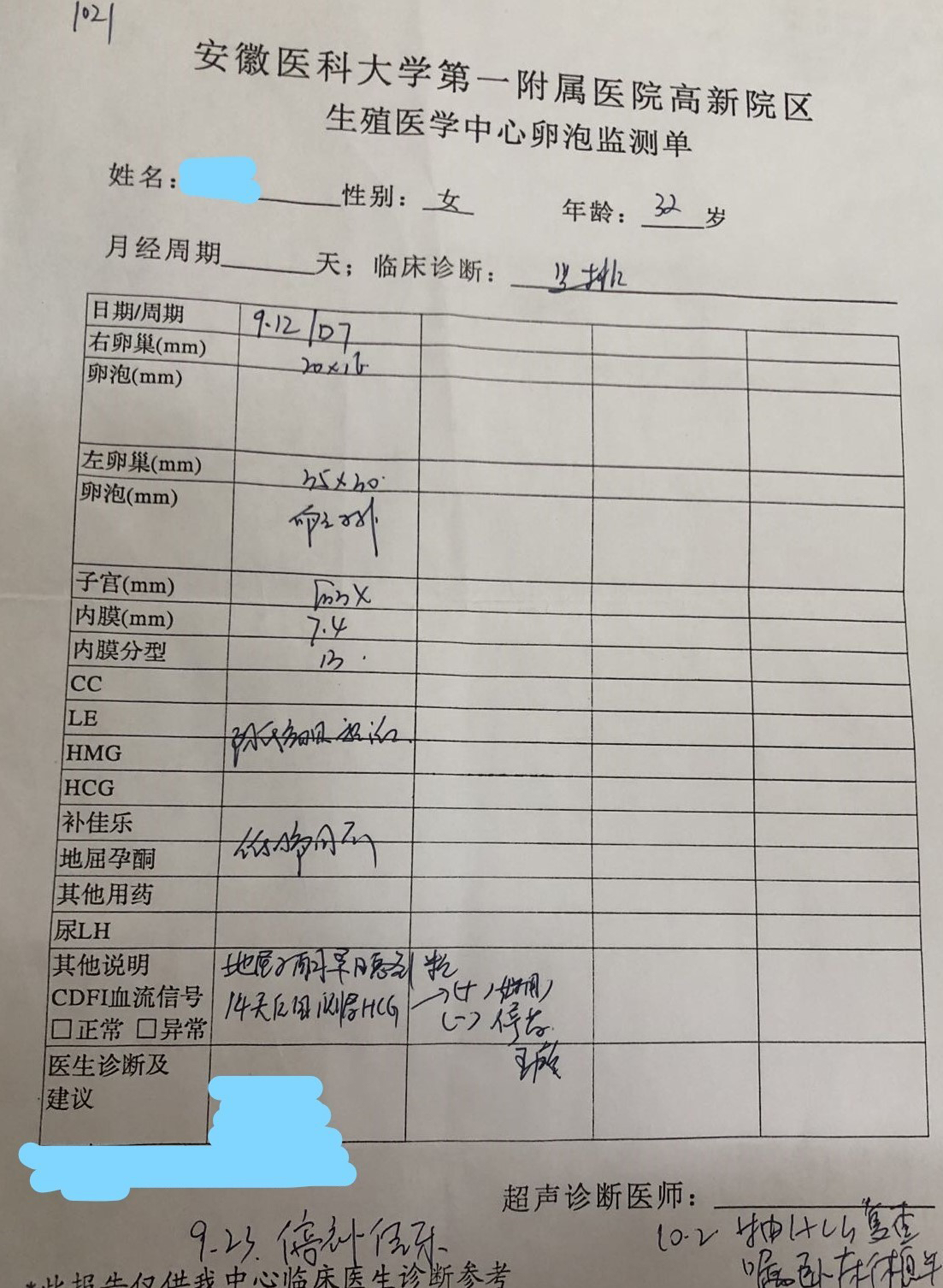

Supplement: Supplementary Figure 2 — The original record indicated the follicle ovulated on day 7 of the menstruation. [file Image_2.JPEG]
